# Effects of lower limb biomechanical characteristics on jump performance in female volleyball players based on long Stretch–Shortening cycle movements

**DOI:** 10.3389/fbioe.2025.1653751

**Published:** 2025-09-18

**Authors:** Beiwang Deng, Yueming Li, Gesheng Lin, Ruixiang Yan, Jianxin He, Duanying Li, Jian Sun

**Affiliations:** ^1^ School of Athletic Training, Guangzhou Sport University, Guangzhou, Guangdong, China; ^2^ Guangdong Provincial Key Laboratory of Human Sports Performance Science, Guangzhou Sport University, Guangzhou, Guangdong, China; ^3^ Badminton Technical and Tactical Analysis and Diagnostic Laboratory, Guangzhou Sport University, Guangzhou, Guangdong, China

**Keywords:** long stretch-shortening cycle, depth jump, propulsion velocity, ankle torque, lower-limb stiffness, volleyball

## Abstract

**Background:**

In volleyball, certain maneuvers (e.g., depth jumps) involve a long stretch-shortening cycle (long-SSC) characterized by a prolonged landing-to-takeoff phase (ground contact time ≥222 ms). However, the key biomechanical factors influencing jump height in such long-SSC movements remain unclear, particularly in female athletes. This study investigated depth jump biomechanics in female volleyball players to identify performance-related factors and inform training optimization.

**Methods:**

Eighteen trained female volleyball players performed maximal-effort depth jumps under 3D motion capture. Pearson correlation analysis examined relationships between biomechanical variables and jump height. Participants were then divided into high (HJG) and low (LJG) jump-height groups based on a median split and compared using independent samples *t*-tests.

**Results:**

Jump height correlated positively with peak propulsion velocity, peak propulsion power, knee flexion-extension angle, peak ankle moment, and peak propulsion impulse (all *p* < 0.05). Compared with LJG, HJG exhibited significantly greater jump height, propulsion velocity, knee flexion-extension angle, and ankle moment but lower leg stiffness and braking force. Differences in contact time, propulsion impulse, and hip angle had moderate effect sizes.

**Conclusion:**

Peak propulsion velocity was the strongest correlate of jump height in long-SSC depth jumps. Propulsion-phase variables, particularly ankle torque and impulse, were more influential than braking-phase variables. In contrast to short-SSC tasks, high lower-limb stiffness appears to provide limited benefit for maximizing performance in long-SSC movements. Training for female volleyball players should therefore prioritize developing propulsion-phase power and ankle strength for these types of jumps.

**Trial registration number:**

ChiCTR2400094392; Registration date: 22/12/2024.

## Highlights

What are the main findings?

- Peak propulsion velocity is the strongest predictor of depth jump height.
- Ankle joint torque correlates more with jump height than hip or knee moments.

What is the implication of the main finding?

- Training should prioritise propulsion-phase power, impulse, and ankle strength.
- Leg-stiffness emphasis can be reduced when targeting long-SSC jump tasks.

## 1 Introduction

Vertical jump performance, often quantified by jump height, is a key determinant of success in volleyball and is determined by a complex interplay of biomechanical factors during the braking and propulsion phases, including impulse generation and power output (Schmidt, 2015). Scoring points and preventing opponent points often hinge on a player’s ability to execute high jumps for attacks and blocks near the net. In fact, actions such as spiking and blocking–which inherently involve vertical jumps–have a disproportionately large impact on match outcomes in high-level play (Afonso and Mesquita, 2011). Prior analyses have shown that players’ anthropometric attributes (height and reach) and *especially* their vertical jump capabilities strongly influence attacking and blocking effectiveness, underscoring the importance of jump training in this sport (Ziv and Lidor, 2010). Coaches and scientists alike have therefore long focused on improving jump performance in volleyball athletes as a means to enhance competitive success (Marshall and Moran, 2015).

High-level female volleyball players perform a substantial number of jumps during competition, which highlights the physical demand and the need for efficient jump mechanics. On average, an elite female player executes on the order of 20–30 jumps per set (Lima et al., 2019). For example, Tillman et al. reported roughly 22 jump-landings per set in high-level women’s volleyball (Tillman et al., 2004). Many of these jumps occur in sequences or in “transition” scenarios rather than in isolation. A middle blocker might perform multiple block jumps in quick succession during a single rally, while an outside hitter often transitions from a defensive action (e.g., a block or dig) immediately into an approach jump for a counter-attack. Match analyses confirm the prevalence of such scenarios; in one study of elite women’s play, outside hitters and opposites engaged in approach runs leading to jump attacks in approximately 40% of their movement sequences during rallies (Rebelo et al., 2022). These types of actions are characterized by a rapid change from landing to take-off (as in repeated blocks) or a longer, deliberate preparatory countermovement (as in transition attacks after defense). Both cases place heavy reliance on the muscle’s stretch–shortening cycle (SSC) behavior. The SSC refers to the physiological mechanism by which an active eccentric muscle contraction (pre-stretch) immediately precedes a concentric contraction, enhancing force and power output via stored elastic energy and the development of reflex-driven reactive strength. In volleyball jumping, use of the SSC (as in a countermovement jump) enables players to achieve greater jump height than a purely concentric effort from a static squat position (Komi and Gollhofer, 1997). Efficient exploitation of the SSC is thus critical for maximizing jump height and reducing energy cost in repeated jumping scenarios relevant to volleyball.

Stretch–shortening cycle (SSC) movements have historically been classified as ‘fast’ (ground contact time, GCT < 250 ms) or ‘slow’ (GCT > 250 ms) based on seminal work. However, following Ünver et al. (2024), we adopt a data-driven three-tier scheme: short-SSC (GCT < 188 ms), mid-SSC (188 ≤ GCT < 222 ms), and long-SSC (GCT ≥ 222 ms). Short-SSC actions, such as rapid block rebounds, exploit high reactive strength and very brief coupling times to leverage short-latency stretch reflex responses and tendon elastic recoil. Mid-SSC covers typical rebound jumps with moderate coupling, and long-SSC tasks, such as an approach spike jump during transition, allow deeper joint flexion and greater force development during the eccentric phase. Most natural volleyball jumps fall along a spectrum between these extremes. For instance, a quick rebound jump with minimal knee bend (such as an immediate second block jump right after landing) constitutes a short-SSC action, whereas a countermovement spike jump with a pronounced knee bend and arm swing would be classified as Long-SSC action. The three-tier framework provides greater granularity for interpreting phase-specific contributions of braking- and propulsion-phase impulses, peak velocities, and joint moments. In practical terms, a volleyball player must rely on reactive strength to rapidly transition from eccentric to concentric action in short-SSC tasks—enabling explosive blocks—while also harnessing the greater propulsion-phase impulse of long-SSC movements when time allows a deeper countermovement, as in well-timed spike jumps. An athlete’s jump performance in volleyball thus depends on both SSC regimes, and training programs must develop reactive strength as well as maximal power production (Pedley et al., 2017).

Plyometric jump drills are commonly used to target these SSC characteristics. In particular, drop jumps and depth jumps are two related plyometric tasks often employed to develop explosive leg power. Although these terms are sometimes used interchangeably, they refer to distinct jump modalities (Clutch et al., 1983). *Drop jump* usually denotes the short-SSC variant: the athlete drops off a box and immediately rebounds upward “as quickly as possible” upon ground contact, minimizing knee flexion and contact time. Performance in a drop jump is typically assessed using the Reactive Strength Index (RSI), defined as jump height divided by ground contact time, with higher values indicating superior explosive reactive ability (Clutch et al., 1983; Jarvis et al., 2022). In contrast, *depth jump* (sometimes called a countermovement depth jump) involves dropping from a height and then using a deeper triple-joint countermovement—greater flexion at the hip, knee, and ankle—to generate maximal jump height (Pedley et al., 2017). In depth jumps, the athlete is allowed a longer coupling time on the ground (≥ 222 ms, a long‐SSC action) in order to maximize concentric force and jump height, even if the contact is not exceedingly brief. Although this extended contact provides more time, the deeper joint excursion and higher eccentric load demand finely timed neuromuscular coordination to decelerate and reverse the downward momentum, engaging the leg extensors through a greater range of motion (Pedley et al., 2017; Taube et al., 2012). Depth jumps thus are characterized by a larger active braking peak and greater positive impulse, emphasizing muscular power output, whereas drop jumps emphasize neural reflexes and lower-limb stiffness. From a training perspective, the drop jump is typically used to develop reactive (short-SSC) power and improve an athlete’s ability to produce force rapidly, while the depth jump is used to develop maximal explosive power and take-off strength in a jumping movement (McBride et al., 2008; Komi and Bosco, 1978). Both jump types address key SSC demands in volleyball: short-SSC (drop jumps) replicate the rapid stretch–reflex needed for successive block rebounds, while long-SSC (depth jumps) mirror the deeper countermovement and extended ground contact of approach attacks and transition plays.

Notably, despite the importance of long-SSC capabilities in volleyball, most biomechanical research on jumping in this population has concentrated on standard countermovement or spike jumps (Ziv and Lidor, 2010). A recent systematic review highlighted a significant gender-representation bias in sports biomechanics; for instance, a 2024 meta-analysis in the Journal of Biomechanics revealed that only 8% of studies were conducted exclusively on female cohorts, while male-only investigations outnumbered female-only ones by more than fivefold (van der Kruk, 2025). This gap is particularly evident for long-SSC movements like depth jumps in female volleyball players. This lack of specific data is problematic, as it leaves critical questions unanswered: Which biomechanical mechanisms (e.g., joint-specific contributions, the role of limb stiffness) differentiate high and low performers in long-SSC jumps? How do these mechanisms differ from the well-studied short-SSC jumps? Without this knowledge, training programs may be sub-optimally designed, potentially limiting performance gains or even increasing injury risk by failing to address the specific demands of long-SSC actions prevalent in women’s volleyball, such as approach spike jumps during transition.

Therefore, the purpose of this study was to investigate long-SSC jump performance in female volleyball players by analyzing the depth jump. Specifically, we aimed to (Schmidt, 2015) characterize the biomechanical profile (kinematics and kinetics) of a maximal depth jump in female volleyball athletes, and (Afonso and Mesquita, 2011) identify the key biomechanical variables that correlate with and differentiate high versus low jump performance. By addressing these questions, this work targets the identified research gap and seeks to provide insight into how female players execute long-SSC movements. Ultimately, characterizing long-SSC (depth jump) behavior can guide the development of more targeted training protocols tailored to the specific demands of women’s volleyball. By addressing these questions, this work targets the identified research gap and seeks to provide insight into how female players handle long-SSC movements. The findings will help clarify whether depth jumps elicit unique movement patterns or performance differences in women, and how this knowledge can be applied to training or injury prevention for volleyball and related sports. Ultimately, distinguishing between short-SSC, mid-SSC, and long-SSC jump mechanics in female athletes—and especially characterizing long-SSC (depth jump) behavior—can guide development of more targeted training protocols tailored to the specific demands of women’s volleyball.

## 2 Research methods

### 2.1 Experimental approach to the problem

This study employed a cross-sectional experimental design to analyze the lower limb biomechanical characteristics of female volleyball players during Depth Jumps and examine their relationship with jump height. Additionally, biomechanical differences between athletes with varying jump heights were compared to provide scientific insights for optimizing jump training.

After familiarizing themselves with the experimental procedures and completing a preliminary test, 18 female volleyball players performed Depth Jump tests over 3 days. During the experiment, lower limb biomechanical data and jump height measurements were collected simultaneously and later standardized during data processing. Correlation analysis was conducted to identify key biomechanical factors influencing jump performance.

To further investigate biomechanical differences based on jump height, athletes were ranked according to their jump performance. The top nine performers were assigned to the High Jump Group (HJG), while the bottom nine were classified into the Low Jump Group (LJG). Key biomechanical parameters were then compared between the two groups to elucidate the biomechanical mechanisms underlying differences in jump height.

### 2.2 Participants

**FIGURE 1:**
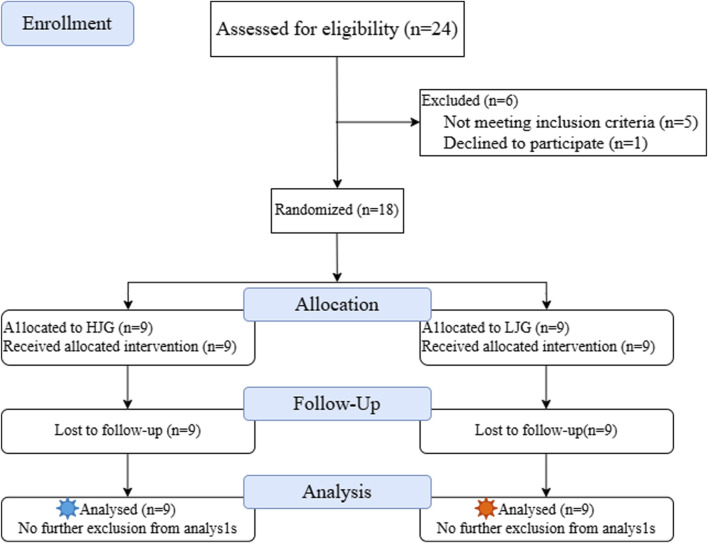
Flow diagram of participant recruitment, exclusion, group allocation, and analysis.

Eighteen collegiate female volleyball players were recruited for this study based on the following inclusion criteria: Inclusion criteria were (Schmidt, 2015): at least 2 years of volleyball-specific training and experience in both unilateral and bilateral resistance training (Afonso and Mesquita, 2011); participation in regional-level volleyball competitions and at least one provincial-level or higher university volleyball league (Ziv and Lidor, 2010); participation in at least two volleyball-specific training sessions per week (Marshall and Moran, 2015); no history of lower limb orthopedic injuries, other lower limb conditions, or cardiovascular diseases in the 6 months prior to the experiment. All participants were members of the same university volleyball team and regularly competed in regional-level tournaments, including the B subgroup of the Elite Division in the Guangdong Collegiate Volleyball League. They were also selected to participate in the 12th Guangdong University Games, a provincial competition held every 4 years, representing a relatively high level of athletic performance among Chinese collegiate athletes. The recruitment, exclusion, group allocation, and analysis of participants are shown in Figure 1.

No significant differences were observed between the HJG and LJG in age, height, weight, or training experience. The participants’ basic characteristics are presented in Table 1. All participants were fully informed of potential risks before testing and provided written informed consent. This study adhered strictly to the ethical principles of the Declaration of Helsinki and received approval from the Ethics Committee of Guangzhou Sport University (Approval No.: 2024LCLL-116). Additionally, the study was retrospectively registered in the Chinese Clinical Trial Registry (Registration No.: ChiCTR2400094392; date of registration: 22/12/2024).

**TABLE 1:** Participant characteristics.

Basic information	HJG (n = 9)	LJG (n = 9)	Total (n = 18)
Age (years)	19.11 ± 0.78	19.22 ± 0.83	19.17 ± 0.79
Height (m)	172.67 ± 4.82	172.44 ± 6.23	172.56 ± 5.40
Weight (kg)	64.93 ± 7.44	64.31 ± 7.09	64.62 ± 7.06
Training Years (years)	6.11 ± 2.52	5.28 ± 2.44	5.69 ± 2.44

### 2.3 Measurements and procedures

Laboratory preparation involved initializing and calibrating the Vicon 3D motion capture system and warming up and calibrating the force platforms to ensure all experimental equipment functioned properly. The motion capture system comprised ten cameras (Arqus, Qualisys, Gothenburg, Sweden) operating at a 200 Hz sampling rate to record three-dimensional kinematic data. Two force platforms (FP4060-10, Bertec, Columbus, United States of America) were set to a 1,000 Hz sampling rate to ensure high-resolution force data acquisition.

Upon arrival at the laboratory, participants underwent height and weight measurements before changing into sportswear. They then performed a standardized 15–20 min warm-up, consisting of foam rolling, jogging, dynamic stretching, neural activation drills, and movement integration exercises. Foam rolling included one set of pressure on major muscle groups using a foam roller or fascia ball, targeting areas such as the quads, hamstrings, calves, back, etc. Dynamic stretching included one set of exercises such as the book-opening stretch, knee-to-chest with calf raise, cradle knee hug, and the “greatest stretch” (a dynamic stretch targeting the hip flexors, hamstrings, and glutes). Neural activation drills consisted of one set of quick high knees and short-distance sprints to engage the nervous system for optimal performance. Movement integration exercises included A skips, a drill to help integrate lower body movement patterns. Following the warm-up, reflective markers were placed on specific anatomical landmarks according to the Cast model. To maintain data collection consistency, the same researcher applied markers to identical body regions across all participants. Height and weight were measured again to facilitate kinematic data standardization. Participants’ clothing and footwear were checked and adjusted to ensure a snug fit, minimizing marker displacement and preventing occlusion. Shoes had to be comfortable and non-restrictive to avoid interference with testing.

For the Depth Jump test, participants stood with their hands on their hips on a 40 cm-high jump platform. For the Depth Jump test, participants stood with their hands on their hips on a jump platform. Upon the ‘Start’ command, they lifted their dominant leg and placed it outside the platform, with the other foot remaining on the platform. They then leaned forward naturally to initiate a free fall, landing on the force platforms positioned beneath each foot, one on the left and one on the right. Participants were instructed to land on the platform as accurately as possible to ensure accurate measurement of ground reaction forces. Experimenters provided verbal encouragement, such as “Jump as high as possible,” to maximize effort. Ground contact time and countermovement depth were not restricted during the test. Each participant completed at least five valid trials at each drop height condition. Each participant completed at least five valid trials, with a 2-min rest interval between each trial to prevent fatigue.

**FIGURE 2:**
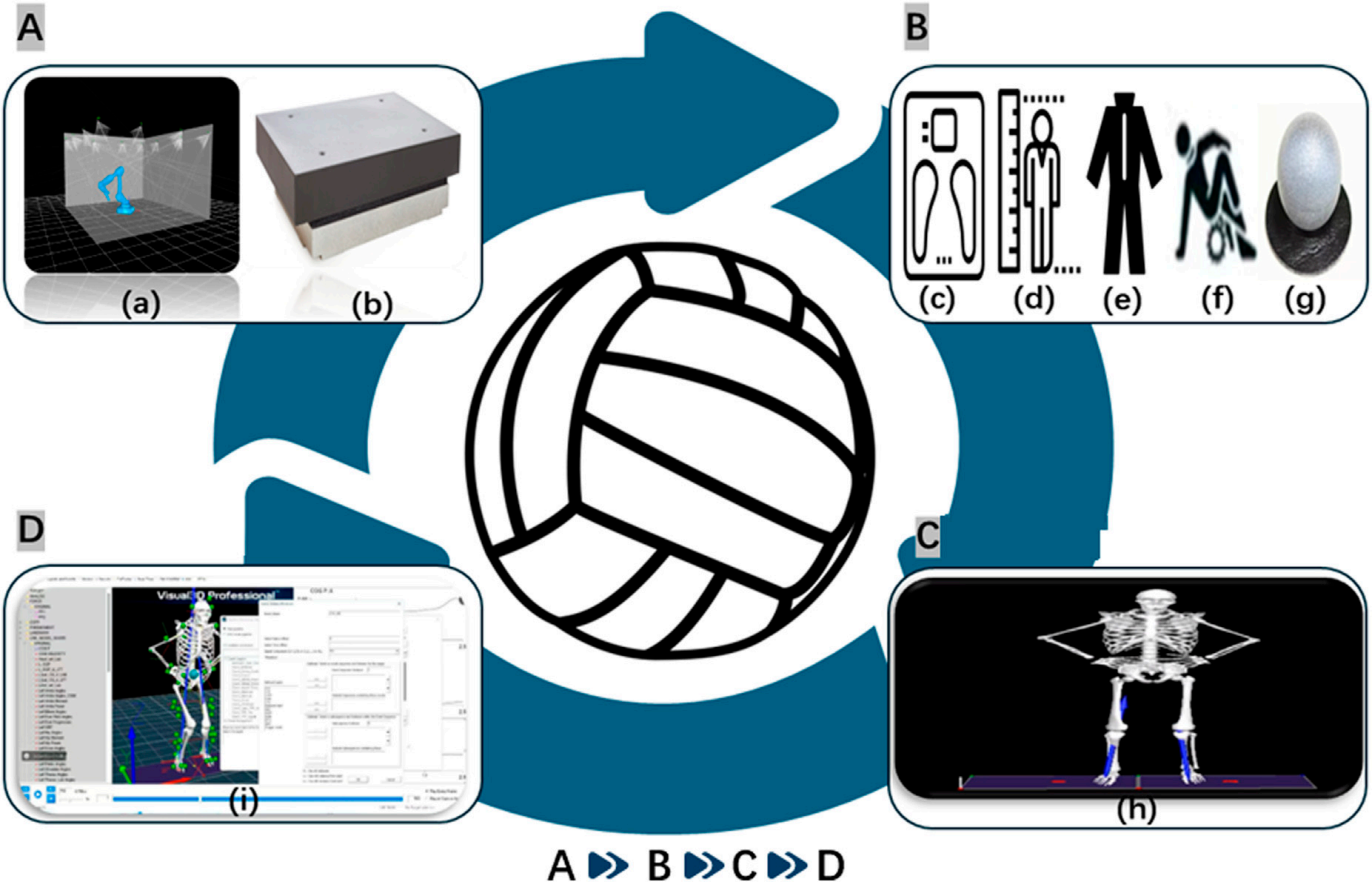
Experimental testing flowchart. Note: **(A)** Equipment calibration (a: Vicon 3D Motion Capture System; b: Force Plate Calibration); **(B)** Participant preparation (c: Change of Clothes; d: Warm-up; e: Marker Placement; f: Body Weight Measurement; g: Height Measurement, etc.); **(C**) Data analysis (h: Drop Jump Data Collection); **(D)** Data Acquisition (i: Visual 3D Data Processing).

A trial was considered valid if it met the following criteria:The center of mass exhibited no obvious upward or forward movement when stepping off the platform. Both feet landed simultaneously, followed by a maximal-effort vertical jump while maintaining hands on hips throughout the movement. During flight, the hip and knee joints extended naturally. The detailed testing procedure is illustrated in Figure 2.

### 2.4 Phase definition

**FIGURE 3:**
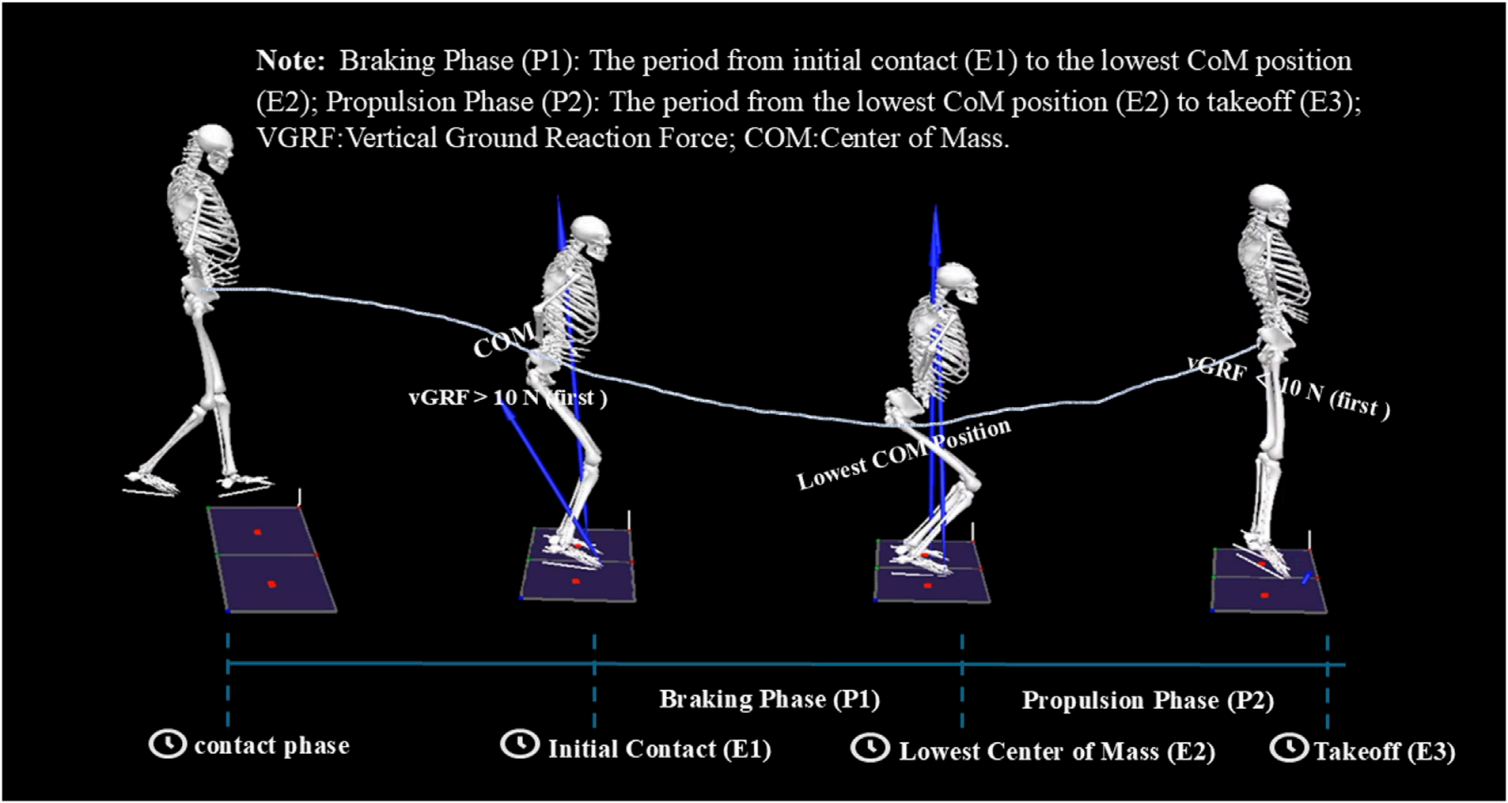
Time events and phase division of the depth jump movement.

Following the established protocol outlined in previous studies (Healy et al., 2018), this study segmented the Depth Jump movement into three events and two phases using vertical ground-reaction force (vGRF) criteria (Figure 3).

Initial contact (E1): The moment when vGRF first exceeds 10 N upon landing, marking the first foot contact with the ground. Lowest center of mass (E2): The moment when the center of mass (CoM) reaches its lowest position, representing the deepest squat position during the movement. Takeoff (E3): The moment when vGRF first drops below 10 N after landing, indicating foot takeoff from the ground.

The depth jump was further divided into two distinct phases: Braking phase (P1): The period from initial contact (E1) to the lowest CoM position (E2). Propulsion phase (P2): The period from the lowest CoM position (E2) to takeoff (E3).

### 2.5 Variable selection, coordinate system, and joint angle definitions

This study defined the joint coordinate system and joint angles using Visual 3D. The joint coordinate system was established based on three anatomical axes of the body: the coronal axis (X-axis, flexion/extension), the sagittal axis (Y-axis, abduction/adduction), and the vertical axis (Z-axis, internal/external rotation). Joint angles were defined as follows: the ankle joint angle was measured between the foot and the extended line of the lower leg, the knee joint angle was defined as the angle between the lower leg and the extended line of the thigh, and the hip joint angle was determined as the angle between the thigh and the extended line of the trunk.

The drop jump is a commonly used movement model for analyzing reactive forces, ground impact, and energy conversion. In drop jump research, variables such as ground reaction force, power, Vertical stiffness, joint torque, joint stiffness, joint angles, and positive/negative work are used to characterize an athlete’s mechanical response during the landing and push-off phasse (Horita et al., 2002). Although this study focuses on the depth jump, certain drop jump parameters were referenced due to their biomechanical similarities.

The kinematic parameters analyzed in this study included jump height, RSI, peak propulsion velocity, and the maximum flexion-extension angles of the hip, knee, and ankle joints during both the braking and propulsion phases. The kinetic parameters included peak braking force, peak propulsion force, peak braking power, peak propulsion power, lower limb stiffness, work performed during the braking and propulsion phases, peak braking impulse, and peak propulsion impulse. The temporal parameter examined in this study was contact time. The calculation methods for the jump height and dynamic indicators in the drop jump test are provided in Table 2. The reliability and variability (ICC and CV) of all measured variables are presented in Supplementary Table S1.

**TABLE 2:** Calculation methods for performance, kinematic, and kinetic variables in the Depth Jump (long-SSC).

Indicator (unit)	Calculation
Jump height (m)	Either (a) h = CoM_peak (air) − CoM_standing
Peak propulsion velocity (m·s^-1^)	Maximum vertical CoM velocity during the propulsion phase P2 (E2→E3)
Contact time (s)	Ground contact duration E1→E3 (IC to TO) using vGRF 10 N thresholds
Impulse during the braking phase (N·s·kg^-1^)	∫[(Fz − m·g)/m] dt over P1 (E1→E2)
Impulse during the propulsion phase (N·s·kg^-1^)	∫[(Fz − m·g)/m] dt over P2 (E2→E3)
Peak force during the braking phase (N·kg^-1^)	Maximum vertical GRF normalized to body mass within P1 (E1→E2)
Peak force during the propulsion phase (N·kg^-1^)	Maximum vertical GRF normalized to body mass within P2 (E2→E3)
Peak power during the braking phase (W·kg^-1^)	Maximum CoM mechanical power per unit mass P/m, where P = (Fz−m·g)·v_CoM, within P1 (E1→E2)
Peak power during the propulsion phase (W·kg^-1^)	Maximum CoM mechanical power per unit mass P/m within P2 (E2→E3)
Energy storage (J·kg^-1^)	Negative CoM work per unit mass W^−^ = ∫(P/m) dt over P1 (E1→E2
Energy release (J·kg^-1^)	Positive CoM work per unit mass W^+^ = ∫(P/m) dt over P2 (E2→E3)
Vertical (leg) stiffness (N·m^-1^·kg^-1^)	k_vert = (Fz_peak − m·g)/Δy_CoM, where Δy_CoM is CoM vertical displacement during P1 (E1→E2); normalized to body mass
Maximum hip flexion–extension angle during the braking phase (°)	Visual3D JCS hip flex-ext DOF; peak value within P1 (E1→E2)
Maximum hip flexion–extension angle during the propulsion phase (°)	Visual3D JCS hip flex-ext DOF; peak value within P2 (E2→E3)
Maximum knee flexion–extension angle during the braking phase (°)	Visual3D JCS knee flex-ext DOF; peak value within P1 (E1→E2)
Maximum knee flexion–extension angle during the propulsion phase (°)	Visual3D JCS knee flex-ext DOF; peak value within P2 (E2→E3)
Maximum ankle flexion–extension angle during the braking phase (°)	Visual3D JCS ankle dorsiflex-plantarflex DOF; peak value within P1 (E1→E2)
Maximum ankle flexion–extension angle during the propulsion phase (°)	Visual3D JCS ankle dorsiflex-plantarflex DOF; peak value within P2 (E2→E3)
Peak hip joint moment during the braking phase (N·m·kg^-1^)	Visual3D inverse-dynamics net internal hip moment (flex-ext); peak magnitude within P1 (E1→E2); normalized to body mass
Peak hip joint moment during the propulsion phase (N·m·kg^-1^)	Visual3D inverse-dynamics net internal hip moment (flex-ext); peak magnitude within P2 (E2→E3); normalized to body mass
Peak knee joint moment during the braking phase (N·m·kg^-1^)	Visual3D inverse-dynamics net internal knee moment (flex-ext); peak magnitude within P1 (E1→E2); normalized to body mass
Peak knee joint moment in the propulsion phase (N·m·kg^-1^)	Visual3D inverse-dynamics net internal knee moment (flex-ext); peak magnitude within P2 (E2→E3); normalized to body mass
Peak ankle joint moment during the braking phase (N·m·kg^-1^)	Visual3D inverse-dynamics net internal ankle moment (dorsi/plantarflex); peak magnitude within P1 (E1→E2); normalized to body mass
Peak ankle joint moment during the propulsion phase (N·m·kg^-1^)	Visual3D inverse-dynamics net internal ankle moment (dorsi/plantarflex); peak magnitude within P2 (E2→E3); normalized to body mass
RSI	Reactive Strength Index = jump height/ground contact time (E1→E3). Report as m·s^-1^ or unitless consistently

(1) Event definitions: E1 (IC) = first frame where vGRF, exceeds 10 N after drop; E2 (min-CoM) = lowest CoM position; E3 (TO) = first frame where vGRF, falls below 10 N after landing. (2) Phases: P1 (Braking) = E1→E2; P2 (Propulsion) = E2→E3. (3) Software: Visual3D (C-Motion). Joint angles (JCS) and net internal joint moments (Newton–Euler inverse dynamics) reported per Visual3D conventions. (4) Normalization: Forces/impulses/powers/joint moments normalized to body mass (kg). Net force uses (Fz − m·g). (5) Filtering: 4th-order zero-lag Butterworth low-pass filter (cutoff 15 Hz) applied to kinematics and GRF, before inverse dynamics and outcome extraction. (6) Integration: Trapezoidal numerical integration at native sampling rates (kinematics 200 Hz; GRF, 1000 Hz).

### 2.6 Data processing

From each participant’s experimental data, five completed jump trials were selected. Marker localization and data interpolation were performed using Qualisys software, and the processed data were exported as C3D files. These files were then imported into Visual 3D for further analysis, where a full-body model was used to establish static modeling and compute relevant biomechanical variables.

Kinematic and kinetic data were smoothed using a fourth-order recursive Butterworth low-pass filter with a 15 Hz cutoff frequency to reduce high-frequency noise. This cutoff frequency was selected based on previous studies that have analyzed high-impact plyometric movements (Khuu et al., 2015) and was confirmed via a residual analysis to ensure that signal power was preserved while minimizing noise (Khuu et al., 2015). To account for inter-individual weight differences, all kinetic data were normalized to body weight. Among the five trials, the three best-performing jumps were selected for analysis, and the mean values of the relevant kinematic and kinetic variables were calculated.

## 3 Statistical analysis

A *post hoc* power analysis was conducted using G*Power (version 3.1.9.7) to determine the statistical power of our sample size (n = 18). For the independent samples t-test, assuming a large effect size (Cohen’s d = 0.8) and an alpha level of 0.05, the achieved power (1-β) was 0.49. For the correlation analysis, assuming a large effect size (ρ = 0.5) and an alpha level of 0.05, the achieved power was 0.73. While the power for the t-test was modest, these analyses provide a quantitative assessment of our findings and can inform sample size calculations for future studies. All variables were computed using Visual 3D, and statistical analyses were conducted using JASP 0.18.3.0. Participants were ranked based on jump height and divided into HJG and LJG using the median split method. Before conducting correlation analysis, normality was assessed for each variable using the Shapiro–Wilk test. Data that did not follow a normal distribution were Box-Cox transformed (Sakia, 1992), ensuring that all transformed variables remained positive. Within-session reliability was evaluated using the coefficient of variation (CV) and the intraclass correlation coefficient (ICC) with a 95% confidence interval. ICCs were calculated in JASP (v0.18.3) using a two-way mixed-effects model with absolute agreement and single-measurement units [ICC(3,1)], based on three within-session depth-jump trials per participant. CVs were computed as the ratio of the standard deviation to the mean from the same three baseline trials, expressed as a percentage (Hopkins, 2000). ICC values were interpreted for relative reliability (Weir and Vincent, 2021): values between 0.5 and 0.75 indicated moderate reliability, values between 0.75 and 0.9 indicated good reliability, and values over 0.90 indicated excellent reliability (Portney and Watkins, 2009). Previous reliability studies reported that biomechanical variables with a CV around 10% are reliable, hence a CV ≤ 10% was set as the standard for declaring variable reliability (Augustsson et al., 2006; Cormack et al., 2008). Pearson correlation analysis was used to assess relationships between biomechanical parameters and jump height. Correlation coefficients (r) were classified as follows: very small (r < 0.1), small (0.1 ≤ r < 0.4), moderate (0.4 ≤ r < 0.7), large (0.7 ≤ r < 0.9), and very large (r ≥ 0.9). To compare differences between the two groups, independent sample t-tests were conducted, and Hedges’ g (*g*) was used to estimate effect sizes, categorized as very small (*g* < 0.2), small (0.2 ≤ *g* < 0.5), moderate (0.5 ≤ *g* < 0.8), and large (*g* ≥ 0.8). Statistical significance was set at *p* < 0.05.

## 4 Results

### 4.1 Correlation between jump height and lower limb biomechanical parameters

As shown in Table 3, peak propulsion velocity (r = 0.911, *p* < 0.001), maximum knee flexion-extension angle during the braking phase (r = 0.610, *p* < 0.01), maximum knee flexion-extension angle during the propulsion phase (r = 0.604, *p* < 0.01), peak power during the propulsion phase (r = 0.521, *p* < 0.05), peak ankle moment during the braking phase (r = 0.580, *p* < 0.05), peak ankle moment during the propulsion phase (r = 0.567, *p* < 0.05), and peak propulsion impulse (r = 0.500, *p* < 0.001) were all significantly correlated with jump height (*p* < 0.05). However, no significant correlations were observed between other lower limb biomechanical parameters and jump height (*p* > 0.05).

**TABLE 3:** Correlation between lower limb biomechanical characteristics and jump height.

Parameter	M	SD	Jump height
Peak propulsion velocity (m/s)	2.621	0.100	0.911***
Maximum hip flexion-extension angle during the braking phase (°)	83.468	16.121	0.252
Maximum hip flexion-extension angle during the propulsion phase (°)	83.344	16.150	0.257
Maximum knee flexion-extension angle during the braking phase (°)	96.709	9.547	0.610**
Maximum knee flexion-extension angle during the propulsion phase (°)	96.731	9.496	0.604**
Maximum ankle flexion-extension angle during the braking phase (°)	105.014	5.756	0.194
Maximum ankle flexion-extension angle during the propulsion phase (°)	105.460	5.677	0.199
Peak force during the braking phase (N·kg^-1^)	19.392	4.288	−0.073
Peak force during the propulsion phase (N·kg^-1^)	12.684	1.485	−0.056
Peak power during the braking phase (W·kg^-1^)	41.032	9.740	−0.021
Peak power during the propulsion phase (W·kg^-1^)	25.651	2.85	0.521*
Peak hip joint moment during the braking phase (N·m·kg^-1^)	−1.725	0.393	0.098
Peak hip joint moment during the propulsion phase (N·m·kg-1)	−1.760	0.353	0.114
Peak knee joint moment during the braking phase (N·m·kg^-1^)	1.972	0.301	0.122
Peak knee joint moment during the propulsion phase (N·m·kg^-1^)	1.789	0.309	0.008
Peak ankle joint moment during the braking phase (N·m·kg^-1^)	−1.415	0.207	0.580*
Peak ankle joint moment during the propulsion phase (N·m·kg^-1^)	−1.589	0.169	0.567*
Vertical (leg) stiffness (N·m^-1^·kg^-1^)	71.552	20.868	−0.363
Energy Storage (J·kg-^1^)	1.915	0.336	0.202
Energy Release (J·kg^-1^)	2.094	0.250	0.425
Peak impulse during the braking phase (N·s·kg^-1^)	2.349	0.431	0.388
Peak impulse during the propulsion phase (N·s·kg^-1^)	2.559	0.235	0.500***
RSI	0.880	0.147	−0.149
Contact time(s)	0.472	0.089	0.453

*p < 0.05,**p < 0.01,***p < 0.001.

### 4.2 Comparison of lower limb biomechanical parameters between High Jump Group and Low Jump Group

As shown in Table 4, the HJG exhibited a significantly greater jump height than the LJG (t = 4.247, *p* < 0.001, *g* = 1.907). Additionally, HJG demonstrated significantly higher values in peak propulsion velocity (t = 4.284, *p* < 0.001, *g* = 1.923), maximum knee flexion-extension angle during the braking phase (t = 2.232, *p* = 0.040, *g* = 1.002), maximum knee flexion-extension angle during the propulsion phase (t = 2.228, *p* = 0.041, *g* = 1.000), peak ankle moment during the braking phase (t = 2.317, *p* = 0.044, *g* = 1.040), and peak ankle moment during the propulsion phase (t = 2.933, *p* = 0.010, *g* = 1.316).

**TABLE 4:** Comparison of lower limb biomechanical characteristics between HJG and LJG.

Parameter	HJG	LJG	*t*	*p*	Hedges’ *g*	95% confidence interval of hedges’ g	
						Lower bound	Upper bound
Contact time (s)	0.501 ± 0.084	0.442 ± 0.088	1.459	0.164	0.655	−0.306	1.596
Jump height (m)	0.416 ± 0.013	0.386 ± 0.017	4.247	<0.001	1.907	0.743	3.029
RSI	0.856 ± 0.154	0.904 ± 0.144	−0.679	0.507	−0.305	−1.230	0.630
Peak propulsion velocity (m/s)	2.692 ± 0.051	2.550 ± 0.085	4.284	<0.001	1.923	0.723	3.078
Peak force during the braking phase (N·kg^-1^)	17.002 ± 3.683	21.781 ± 3.558	−2.800	0.013	−1.257	−2.261	−0.221
Peak force during the propulsion phase (N·kg^-1^)	12.088 ± 1.388	13.279 ± 1.400	−1.813	0.089	−0.814	−1.768	0.163
Peak power during the braking phase (W·kg^-1^)	37.764 ± 9.623	44.299 ± 9.217	−1.471	0.161	−0.660	−1.602	0.301
Peak power during the propulsion phase (W·kg^-1^)	26.183 ± 1.726	25.120 ± 3.707	0.780	0.451	0.350	−0.592	1.277
Vertical (leg) stiffness (N·m^-1^·kg^-1^)	58.444 ± 18.037	84.660 ± 14.610	−3.388	0.004	−1.521	−2.570	−0.436
Energy storage (J·kg^-1^)	1.865 ± 0.370	1.965 ± 0.313	−0.624	0.542	−0.280	−1.205	0.653
Energy release (J·kg^-1^)	2.141 ± 0.160	2.048 ± 0.321	0.780	0.451	0.350	−0.592	1.277
Peak impulse during the braking phase (N·s·kg^-1^)	2.353 ± 0.451	2.346 ± 0.437	0.031	0.975	0.014	−0.910	0.938
Peak impulse during the propulsion phase (N·s·kg^-1^)	2.644 ± 0.202	2.474 ± 0.245	1.610	0.128	0.723	−0.246	1.670
Maximum hip flexion-extension angle during the braking phase (°)	87.568 ± 18.185	79.368 ± 13.555	1.233	0.236	0.554	−0.399	1.489
Maximum hip flexion-extension angle during the propulsion phase (°)	87.501 ± 18.221	79.187 ± 13.541	1.241	0.233	0.557	−0.396	1.493
Peak hip joint moment during the braking phase (N·m·kg^-1^)	−1.745 ± 0.366	−1.706 ± 0.440	−0.205	0.840	−0.092	−1.015	0.834
Peak hip joint moment during the propulsion phase (N·m·kg^-1^)	−1.772 ± 0.349	−1.748 ± 0.376	−0.138	0.892	−0.062	−0.985	0.863
Maximum knee flexion-extension angle during the braking phase (°)	100.959 ± 9.878	92.459 ± 7.447	2.232	0.040	1.002	0.002	1.975
Maximum knee flexion-extension angle during the propulsion phase (°)	100.958 ± 9.891	92.505 ± 7.321	2.228	0.041	1.000	4.139 × 10^−4^	1.972
Peak knee joint moment during the braking phase (N·m·kg^-1^)	1.934 ± 0.276	2.010 ± 0.336	−0.523	0.608	−0.235	−1.159	0.697
Peak knee joint moment in the propulsion phase (N·m·kg^-1^)	1.761 ± 0.282	1.818 ± 0.348	−0.383	0.707	−0.172	−1.095	0.757
Maximum ankle flexion-extension angle during the braking phase (°)	104.942 ± 6.016	105.085 ± 5.848	−0.051	0.960	−0.023	−0.947	0.901
Maximum ankle flexion-extension angle during the propulsion phase (°)	105.537 ± 5.817	105.383 ± 5.885	0.056	0.956	0.025	−0.899	0.949
Peak ankle joint moment during the braking phase (N·m·kg^-1^)	−1.314 ± 0.250	−1.516 ± 0.075	2.317	0.044	1.040	−0.015	2.052
Peak ankle joint moment during the propulsion phase (N·m·kg^-1^)	−1.492 ± 0.141	−1.686 ± 0.140	2.933	0.010	1.316	0.271	2.329

Welch’s t-test.

Conversely, lower limb stiffness (t = −3.388, *p* = 0.004, *g* = −1.521) and peak braking force (t = −2.800, *p* = 0.013, *g* = −1.257) were significantly lower in HJG compared to LJG. No significant differences were found between the two groups for other lower limb biomechanical parameters (*p* > 0.05).

Although certain variables did not reach statistical significance, effect size analysis indicated moderate effect sizes for contact time (*g* = 0.655), peak propulsion impulse (*g* = 0.723), maximum hip flexion-extension angle during the braking phase (*g* = 0.554), and maximum hip flexion-extension angle during the propulsion phase (*g* = 0.557), suggesting that these variables still exhibited notable differences between the two groups.

## 5 Discussion

This study aimed to examine the relationship between lower limb biomechanical characteristics and jump height and to compare the biomechanical differences between HJG and LJG to identify key indicators affecting jump performance. Through correlation analysis and group comparisons, this study provides theoretical insights for improving jump performance and optimizing training programs. The findings revealed significant correlations between peak propulsion velocity, peak power during the propulsion phase, knee flexion-extension angles, ankle moments, and peak propulsion impulse with jump height. Additionally, HJG exhibited significantly greater jump height, center of mass propulsion velocity, maximum knee flexion-extension angle, and peak ankle moment compared to LJG, while lower limb stiffness and peak braking force were lower in HJG. Although some variables did not reach statistical significance, effect size analysis indicated that contact time, peak propulsion impulse, and hip flexion-extension angle demonstrated moderate effect sizes, suggesting a potential advantage for HJG in these parameters.

A key finding of this study was the strong positive correlation between peak propulsion velocity and jump height (r = 0.911, *p* < 0.001). Additionally, HJG demonstrated a significantly higher peak propulsion velocity than LJG, further suggesting that peak propulsion velocity may serve as a key performance indicator for jump height in long-SSC depth jumps. This phenomenon is closely related to the eccentric-concentric movement mechanism in jumping. In Depth Jumps, the propulsion (concentric) phase is typically preceded by a countermovement eccentric phase, during which the center of mass accelerates downward, leading to a higher velocity upon ground contact. The SSC plays a critical role in this mechanism: during the eccentric phase, elastic structures within the antagonist muscles are stretched, and in the subsequent concentric phase, the stored elastic energy is effectively released and converted into upward force, thereby increasing the body’s upward take-off velocity and ultimately enhancing jump height. In contrast, in Squat Jumps, where no eccentric phase is involved, jump height also correlates with vertical take-off velocity. However, in the absence of an eccentric phase, jump performance relies primarily on concentric muscle activity, with velocity and resulting jump height being more influenced by neural recruitment capacity rather than energy storage and release mechanisms (Bosco et al., 1981). These findings align with previous studies (Kollias et al., 2001; Yamauchi and Ishii, 2007). For instance, Yamauchi and Ishii (2007), using a countermovement jump, identified peak propulsion velocity as a key determinant of vertical jump height, reinforcing its significance in jump performance. Additionally, Kollias et al. (2001),using a squat jump, found that sprinters exhibited higher maximal force and propulsion velocity in vertical jump tests compared to athletes in other sports, likely due to their ability to generate greater propulsion force and acceleration during the concentric phase, thereby achieving superior vertical jump performance (Ugrinowitsch et al., 2007). The HJG’s ability to achieve a higher peak propulsion velocity is likely a multifactorial outcome rooted in their superior biomechanics observed in this study. Specifically, their significantly greater peak ankle moment during the propulsion phase suggests a more powerful push-off from the distal joint. This, combined with their trend towards a larger propulsion impulse (moderate effect size), indicates a more effective application of force over the duration of the concentric phase. Furthermore, their greater knee flexion angle may have allowed them to utilize a longer range of motion to generate this velocity, effectively translating eccentric loading into concentric power.

The findings showed a strong positive correlation between propulsive impulse and jump height; compared with LJG, HJG exhibited a moderate advantage in propulsive impulse, whereas LJG showed a moderate advantage in peak braking force. Taken together, this pattern indicates that propulsion-phase force–time production, rather than braking force alone, is more consequential for jump height. Consistent with prior work (Aagaard et al., 2002; Abernethy et al., 1995), both average and peak concentric power correlate with jump height. Dowling et al. (Dowling and Vamos, 1993) reported a strong correlation between CMJ height and peak power during the concentric phase (r = 0.928, *p* < 0.01). Similarly, Ashley and Weiss (1994) observed a significant relationship between peak power and vertical jump performance (r = 0.80, *p* < 0.05 to r = 0.83, *p* < 0.01), albeit in Squat Jump tasks. These studies highlight peak power as a critical factor associated with jump height. However, in the present study, while power during the propulsion phase was significantly correlated with Depth Jump height, the correlation was moderate, suggesting that power output alone may not be the strongest correlate of vertical jump height. Since power is the product of force and velocity, and peak power is closely related to velocity (Hermassi et al., 2011), the present results further reinforce the notion that jump performance is strongly associated with peak propulsion velocity. Despite previous findings (Dowling and Vamos, 1993; Ashley and Weiss, 1994) suggesting that the relationship between peak power and jump height is not fully understood, our findings indicate that peak concentric power remains an important indicator of depth-jump performance.

The correlation between peak propulsion impulse and Depth Jump height was significant (r = 0.500, *p* < 0.001), although the correlation coefficient was relatively low. Ferragut et al. (2003) reported that positive impulse explained 77% of CMJ height variance, suggesting that the product of force and its duration is a more reliable predictor of jump height than force applied over a short period. Ferragut et al. (Ugrinowitsch et al., 2007) studied 53 participants, including male and female volleyball players at different levels (national league and university athletes) and physical education students. In the current study, eccentric-phase kinetic variables showed small, non-significant correlations with depth-jump height, consistent with Ferragut et al. (2003). Additionally, LJG demonstrated significantly higher peak braking force than HJG, whereas peak force during the propulsion phase showed no significant correlation with jump height. This suggests that vertical peak force may not be the most critical factor influencing jump performance. Cordova and Armstrong (1996) similarly observed no significant relationship between peak force and jump height. This discrepancy may be attributed to ankle instability and poor movement coordination in LJG. Previous research has indicated that individuals with ankle instability exhibit higher peak vGRF at landing and greater variability in ground contact time (Lin et al., 2022). Poor movement coordination can lead to excessive landing forces and inefficient braking, increasing foot loading at ground contact and ultimately impairing jump performance (Bates et al., 2013).

When athletes aim to maximize jump height, Drop Jump techniques often evolve toward increased countermovement amplitude and prolonged ground contact time, resembling Depth Jump mechanics (Hunter and Marshall, 2002). This study found that peak ankle moments during plantarflexion and dorsiflexion, contact time, and jump height were significantly correlated, with HJG displaying greater ankle moments, longer contact time, and larger knee and hip flexion-extension angles. These findings suggest that enhancing hip and knee extension strength, improving ankle torque production capacity, and increasing contact time may contribute to better Depth Jump performance. Previous studies have shown that a higher center of mass (CoM) at the end of the countermovement shortens the available acceleration distance and is negatively associated with jump height (Wagner et al., 2009). Conversely, a lower CoM and larger joint angles indicate a longer acceleration distance, which can be beneficial for jump height if sufficient impulse is generated. Studies have also found that higher vertical jumps are associated with greater knee and hip flexion angles (Domire and Challis, 2007), further supporting the direct relationship between squat depth and jump performance.

Biomechanically, human limb movement is primarily driven by joint torques generated by muscles, regulated by the neuromuscular system. This study found that, compared to the peak torques of the hip and knee joints, the ankle joint’s peak torque showed a stronger association with depth jump performance. Panoutsakopoulos et al. reported that the ankle joint’s contribution depends on the torque-generating capacity of the plantar flexor muscles (Panoutsakopoulos and Bassa, 2023), which is especially critical in the later phase of rapid SSC movements. Increased ankle joint torque enables faster plantar flexion, shortening the concentric phase and enhancing jump performance (Yoon et al., 2007). Additionally, muscle force output is generally linked to its ‘optimal’ length (Bobbert and Casius, 2005). Deviation from this optimal range reduces force production, potentially impairing movement performance. Beyond muscle length considerations, the ankle joint is highly involved in the final push-off phase, where greater torque may enhance the efficiency of force transfer to the ground. In long-SSC tasks, the extended ground contact time allows for larger ankle moments and increased mechanical work, while the gastrocnemius–Achilles tendon complex serves as a primary elastic element for energy storage and release (Kubo et al., 2000). Efficient utilization of this elastic element depends on minimizing muscle fascicle deformation and maximizing tendon elongation during the braking phase (Ishikawa et al., 2005), thereby improving energy return in the propulsion phase. These biomechanical factors, combined with coordinated hip and knee extension (Fukashiro et al., 2006), may help explain the stronger association between ankle torque and jump height observed in the HJG. Thus, optimizing muscle torque generation and jump performance requires precise adjustments in knee and hip joint angles, as well as contact time.

In drop jumps, muscle activity during both the braking and propulsion phases is crucial for energy storage and release. During the braking phase, muscles and tendons stretch upon landing, similar to a spring’s elongation. Tendons, being more efficient than muscle tissue in storing and releasing energy, benefit from greater stiffness, which allows for faster force transmission to bones and enhances the efficiency of concentric contractions within the muscle-tendon unit (Witvrouw et al., 2007). To optimize energy conversion in the SSC, athletes should focus on minimizing muscle deformation in the braking phase, allowing for greater tendon deformation, which facilitates better energy storage. In contrast, depth jumps with relatively greater countermovement amplitude (Turner and Jeffreys, 2010; Hunter and Marshall, 2002), tend to exhibit higher muscular compliance, which results in more energy being stored in muscle tissue rather than tendons during the braking phase, reducing overall energy conversion efficiency across the involved joints. This lower efficiency in energy conversion means that during the propulsion phase, energy production relies more on active muscle work than on the energy stored in the muscle-tendon unit during the braking phase. Therefore, joint power and work during the propulsion phase are primarily driven by the muscles’ ability to generate force through rapid contractions (Van Der Kruk et al., 2018).

Interestingly, our study found that the HJG had significantly less lower limb stiffness than the LJG. At first glance, this result might seem counterintuitive, since high stiffness is often considered beneficial for explosive performance (Butler et al., 2003). However, the role of stiffness in performance is highly dependent on the task’s GCT. In short-SSC movements (<188 m), high lower-limb stiffness facilitates rapid force transmission to the ground and efficient tendon elastic recoil, thereby supporting a faster rate of force development (Wilson and Flanagan, 2008). Conversely, for tasks involving a long-SSC—such as a maximal depth jump with a much longer contact time—a more compliant (less stiff) lower limb may actually be advantageous (Kalkhoven and Watsford, 2018). A more compliant leg spring allows for a larger and deeper countermovement, which increases the time and range for force development and enables the muscles to generate greater work during the propulsion phase. Our finding aligns with Laffaye et al. (2005), who reported that athletes achieving higher jumps exhibited reduced lower limb stiffness in similar jumping tasks. Therefore, in long-SSC depth jumps, maximizing jump height likely relies more on muscular work output and impulse generation over an extended contact period, rather than on the rapid elastic energy recoil associated with high stiffness.

## 6 Conclusion

This study reveals a close relationship between lower limb biomechanical characteristics and jump performance during SSC movements in female volleyball players. Among the analyzed variables, peak propulsion velocity, force output during the propulsion phase, and ankle joint moment were identified as the primary contributors to depth jump performance. Compared to the braking phase, power and impulse output during the propulsion phase played a more critical role in jump outcomes. Although lower limb stiffness is beneficial for short-SSC movements, it does not appear to be a key mechanism in long-SSC tasks such as depth jumps. These findings suggest that moderately increasing ground contact time, enhancing hip and knee extension strength, and improving ankle joint moment output may contribute to better jump performance in long-SSC movements.

## 7 Limitations and future directions

This work should be viewed in light of several constraints. First, the cohort comprised eighteen trained female athletes from a single collegiate volleyball team, representing a specific competitive context; therefore, generalizability to other age groups or competitive levels may be limited. Second, the cross-sectional design identifies associations but cannot establish causality; only longitudinal training interventions can determine whether modifying factors such as propulsive velocity causally enhance jump height. Third, the use of a median split to form performance groups is a methodological simplification that may obscure the continuous nature of athletic ability. Future research should examine these biomechanics across various drop heights and under more ecologically valid, match-like conditions, potentially incorporating *in vivo* measures (e.g., ultrasonography) to provide a more complete picture of muscle–tendon dynamics.

## Data Availability

The original contributions presented in the study are included in the article/Supplementary Material, further inquiries can be directed to the corresponding authors.

## Abbreviations

SSC, Stretch–Shortening Cycle; RSI, Reactive Strength Index; HJG, High Jump Group; LJG, Low Jump Group; vGRF, Vertical Ground Reaction Force; GCT, Ground Contact Time.
