# Supplementary material for: Effects of lower limb biomechanical characteristics on jump performance in female volleyball players based on long Stretch–Shortening cycle movements
Source: Front Bioeng Biotechnol. 2025 Sep 18;13:1653751. doi: 10.3389/fbioe.2025.1653751 (PMC12489256; doi:10.3389/fbioe.2025.1653751)
Supplement: Supplementary file 1 [file Table1.docx]

Table S1 Reliability and variability analysis of test indicators

| Test indicators | ICC(95%CI) | CV(%) |
| --- | --- | --- |
| Contact time | 0.709 (95% CI [0.596, 0.767]) | 9.51% |
| Jump height | 0.713 (95% CI [0.601, 0.770]) | 3.49% |
| RSI | 0.962 (95% CI [0.601, 0.770]) | 8.45% |
| Peak propulsion velocity | 0.850 (95% CI [0.709, 0.935]) | 3.92% |
| Peak force during the braking phase | 0.985 (95% CI [0.963, 0.995]) | 2.67% |
| Peak force during the propulsion phase | 0.963 (95% CI [0.909, 0.987]) | 2.27% |
| Peak power during the braking phase | 0.906 (95% CI [0.818, 0.953]) | 2.48% |
| Peak power during the propulsion phase | 0.899 (95% CI [0.786, 0.948]) | 2.41% |
| Lower limb stiffness | 0.825 (95% CI [0.607, 0.916]) | 1.23% |
| Energy released during the braking phase | 0.676 (95% CI [0.549, 0.740]) | 9.27% |
| Energy released during the propulsion phase | 0.701 (95% CI [0.584, 0.760]) | 5.92% |
| Peak impulse during the braking phase | 0.780 (95% CI [0.513, 0.892]) | 0.89% |
| Peak impulse during the propulsion phase | 0.660 (95% CI [0.527, 0.727]) | 9.52% |
| Maximum hip flexion-extension angle during the braking phase | 0.625 (95% CI [0.479, 0.699]) | 5.79% |
| Maximum hip flexion-extension angle during the propulsion phase | 0.659 (95% CI [0.526, 0.727]) | 1.42% |
| Peak hip joint moment in the braking phase | 0.966 (95% CI [0.965, 0.967]) | 3.89% |
| Peak hip joint moment in the propulsion phase | 0.959 (95% CI [0.958, 0.961]) | 3.80% |
| Maximum knee flexion-extension angle during the braking phase* | 0.884 (95% CI [0.639, 0.956]) | 0.80% |
| Maximum knee flexion-extension angle during the propulsion phase* | 0.876 (95% CI [0.623, 0.953]) | 0.96% |
| Peak knee joint moment in the braking phase | 0.915 (95% CI [0.807, 0.962]) | 1.48% |
| Peak knee joint moment in the propulsion phase | 0.907 (95% CI [0.816, 0.953]) | 1.55% |
| Maximum ankle flexion-extension angle during the braking phase | 0.859 (95% CI [0.644, 0.936]) | 2.09% |
| Maximum ankle flexion-extension angle during the propulsion phase | 0.852 (95% CI [0.636, 0.932]) | 2.29% |
| Peak ankle joint moment in the braking phase | 0.975 (95% CI [0.948, 0.989]) | 2.32% |
| Peak ankle joint moment in the propulsion phase | 0.940 (95% CI [0.857, 0.977]) | 2.63% |
